# Metabolic and Endocrine Markers of Oregano Essential Oil Effects on Antibacterial Immunity, Reproductive Function, Nutritional Status, and Production Performance of Late-Phase Laying Hens

**DOI:** 10.3390/vetsci12121213

**Published:** 2025-12-18

**Authors:** Samira Hadef, Nawel Lezzar, Mohamed Walid Hamlaoui, Ahmed Hadef

**Affiliations:** 1 Department of Veterinary Sciences, Faculty of Nature and Life Sciences, Chadli Bendjedid University of El Tarf, P.O. Box 73, El-Tarf 36000, Algeria; s.hadef@univ-eltarf.dz; 2 Laboratory of Development and Control of Hospital Pharmaceutical Preparations, Faculty of Medicine, Badji Mokhtar University of Annaba, Annaba 23000, Algeria; 3 Institute of Veterinary Sciences, Frères Mentouri Constantine I University, Constantine 25100, Algeria; nawel.lezzar@umc.edu.dz; 4 Department of Veterinary Sciences, Faculty of Natural and Life Sciences, Earth and Universe Sciences, Mohamed Khider University of Biskra, P.O. Box 145 RP, Biskra 07000, Algeria; mohamedwalid.hamlaoui@univ-biskra.dz

**Keywords:** additives, bacterial infection, egg production, estradiol, hens, metabolic profile, *Origanum*, procalcitonin

## Abstract

This study aimed to evaluate the efficacy of oregano essential oil (OEO) in improving the production performance, health, and welfare of late-phase laying hens raised under commercial farm conditions by analyzing its effect on performance metrics and metabolic and endocrine profiles. Daily performance data for approximately 7884 Hy-Line Brown layers divided into two commercial flocks, one consisting of 96-week-old hens (*n* = 3849) and the other of 79-week-old hens (*n* = 4035), were recorded before (Pre-OEO Tx), during (OEO Tx-Week) and one week (Post-OEO Tx Week) following the week of water supplementation with commercial oregano essential oil (5%) of *Origanum heracleoticum* containing carvacrol (79.75%) as the main component (300 mL of product/1000 L of water). The results show a significant improvement in hen-day egg production (HDEP) during treatment (*p* < 0.05), a significant decrease in daily mortality one week after the cessation of treatment, mainly in the youngest hens (*p* < 0.05), and a reduction in feed conversion rate (*p* < 0.05). The general model (GLM) analysis of data from blood samples collected before and after OEO addition showed a significant decrease in plasma levels of procalcitonin (PCT), calcium, albumin (*p* < 0.05), and aspartate aminotransferase (AST) (*p* < 0.01). In contrast, a significant increase in estradiol, total protein globulin (*p* < 0.01), and phosphorus levels (*p* < 0.05) was recorded. The changes in endocrine profiles were significantly related to a restoration of calcium–phosphorus balance and a decrease in hepatic activity of AST and gamma glutamyl transferase (GGT). These results reveal the investigative value of PCT, in conjunction with metabolic profiling and reproductive hormones, for evaluating the effectiveness of phytogenic additives. Further studies are suggested to determine whether essential oil components can improve health and production performances of laying hens by a potential concurrent modulation of their metabolism, inflammatory response, and reproductive axis function.

## Simple Summary

Evaluating the effectiveness of phytogenic products in combating antibiotic resistance and improving poultry production is crucial for establishing their value under commercial farming conditions. This study aimed to assess the effectiveness of a widely used medicinal plant extract, oregano essential oil (OEO), to enhance egg production and control bacterial disturbance in late-phase laying hens raised into commercial flocks. After OEO water supplementation to aged Hy-Line Brown hens, an improvement in antibacterial immunity and anti-inflammatory response, indicated by a decrease in procalcitonin, an increase in plasma protein levels, and a booster effect on estradiol concentration, was noted. These changes were related to the reestablishment of calcium and phosphorus balance and a decrease in liver enzyme activities, leading to more efficient feed conversion and a slight increase in production performance. This study is the first to report the diagnostic value of procalcitonin to evaluate the antimicrobial efficacy of oregano essential oil. This phytogenic additive seems to be a credible approach supporting one-health and eco-friendly concepts in the poultry sector.

## 1. Introduction

Global egg production has seen a marked evolution in recent years, with a 7% rise between 2019 and 2023, reaching a total of approximately 91.13 million tons of eggs according to FAO data [1]. However, this increase has not affected all countries, as is the case with Algeria, which experienced a notable decline of 4% over the same period, with the production of 0.32 million tons of fresh shell eggs in 2023 [1]. While production enhancement is a major concern for developing countries, the control of antibiotic resistance is a primary concern for developed countries. The need to act on production and health issues (animal and human), while ensuring animal welfare, has led research towards new strategies (intelligent, economical, and safe). These involve, among other things, the use of polyvalent boosters as multifunctional feed additives that can simultaneously improve production performance, health status, and animal welfare, shifting the exploration between two major classes of additives, from probiotics [2,3] to phytogenic eubiotics [4,5,6,7,8]. Thus, the potential of phytogenic additives has prompted researchers in recent years to exploit them to improve production and feed efficiency, stimulate anti-infectious immunity, ensure animal welfare, and provide consumers with microbiologically safe, nutritionally valuable, and nontoxic animal-derived products.

The benefits of using essential oils as a phytogenic eubiotic agent in feed for laying hens have been the subject of several studies [7,9]. The essential oil of species belonging to the *Origanum* genus (OEO) of the Lamiaceae family is known for its high content of the phenolic compounds carvacrol and thymol [10], depending on its geographical origin [11,12]. It is the most widely tested natural extract in laying hens for its ability to improve production levels and health status due to its antimicrobial, immunostimulant, and antioxidant properties [13,14]. Some authors have reported the ability of this aromatic oil to improve production performance by increasing the rate of daily egg production [15], the quality of the egg and the eggshell [16], and digestion through its action on the digestive flora [15]. The effect of this oil on the health and immunity of hens has also been highlighted in a number of studies, which have confirmed the effectiveness of this extract in reducing the number of pathogenic bacteria commonly found in laying hen farms and affecting the hen reproductive system, particularly *E. coli* [15,17] and *Salmonella* [17,18]. Furthermore, the bioactive compounds in this oil, which possess antimicrobial and antioxidant properties, are known for their effects on egg hygienic quality, minimizing the number of bacteria inside and outside (shell) of eggs [19,20], on egg industrial quality, such as the storage duration [21] via its antioxidant potential, and on eggshell quality, including its weight [19,20].

Clearly, the effectiveness of using essential oils as a natural booster must be evaluated using reliable diagnostic methods. Evaluations, both clinical (using production parameters, such as egg production rates, mortality, daily feed intake, average egg weight, and feed conversion ratio, and egg quality parameters) and paraclinical (by establishing the biochemical blood profile of laying hens), are often used for this purpose. These tools have been used jointly as indicators of the effect of anti-stress feed additives during heat stress (utility of glucose and cholesterol testing) caused by low or high temperatures in laying hens [22] and to assess the effect of medicinal plant extracts on bacterial infections, such as *E. coli 078* (relevance of ALT, AST, LDH, ALP, globulin, total protein, and albumin) in broiler chickens only [23]. Although these biomarkers have been employed to evaluate essential oils in broilers and heat stress in layers, a comprehensive assessment integrating metabolic profiles with endocrine and inflammatory markers (particularly procalcitonin) remains absent for commercial late-phase laying hens receiving OEO supplementation. Furthermore, these indicators have also been used to verify the effectiveness of antibiotherapy or the substitution of an antibiotic (feed antibiotic) with a feed additive such as probiotics (cholesterol, triglycerides, and progesterone levels) in laying hens [24]. Recently, some blood biomarkers have been used to evaluate, in Hy-Line Brown layers reared under experimental conditions during the peak laying phase, the effect of commercial oregano essential oil on the lipid profile (cholesterol, triglycerides, and high-density lipoproteins), liver indices (AST, ALT), and antioxidant profile as indicators of health status [25]. Thus, previous studies have focused primarily on peak production phases under controlled conditions, leaving a critical knowledge gap regarding OEO efficacy during the commercially challenging late-production period when hens face heightened metabolic demands and immunological vulnerability.

The use of endocrine function exploration through hormone testing, including reproductive hormones (estradiol, follicle-stimulating hormone, luteinizing hormone, and progesterone), has also been adopted to explain the effects of essential oils on production performance in hens at early and late phases of the production cycle [26]. In human medicine, a prohormone called procalcitonin (a precursor of calcitonin) has proven to be an important tool for the rapid and early diagnosis of bacterial infection [27] and for guiding antibiotic use [28]. However, its use in veterinary medicine has been limited to research purposes in certain species (equine, bovine, and canine) due to the lack of validated commercial kits for veterinary species [29]. Recently, in poultry, it has been used to monitor the response of broiler chickens, given arginine as a dietary supplement, to intestinal inflammation following infection with *S. typhimurium* [30] or *C. perfringens* [31]. Given its scarcity in poultry research, the use of procalcitonin to assess the efficacy of phytogenic additives in laying hens can be a new pioneering approach to evaluating the antimicrobial and anti-inflammatory properties of essential oils in commercial poultry production.

This study aims to evaluate the effectiveness of oregano essential oil supplementation by establishing comprehensive metabolic and hormonal profiles, including the novel application of procalcitonin as an antimicrobial efficacy biomarker, in commercial laying hens under real-world farming conditions and during the critical “late phase of the production cycle.” During this final period, older hens become more demanding and more sensitive to any zootechnical (feed-related), intrinsic biological (immunity), extrinsic (e.g., bacteria), and environmental disorders, thus influencing the poultry producer’s decision to end or extend their productive career.

## 2. Materials and Methods

### 2.1. Birds, Medical History, and Rearing Conditions

**Figure 1:**
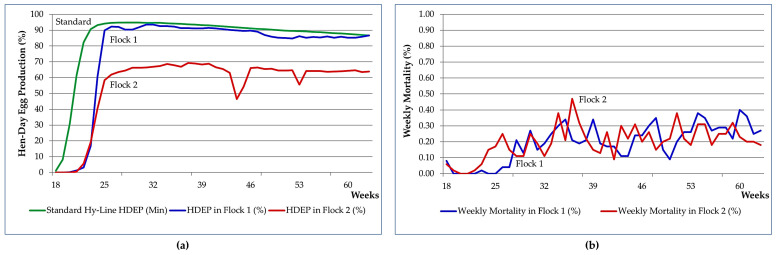
Comparative production history of studied flocks: (**a**) hen-day egg production (HDEP); (**b**) weekly mortality.

This research is a real-world study that was conducted according to a before–after design without a control group for ethical reasons, such as depriving certain cages of the additive without the owner’s consent, and practical constraints related to on-farm conditions, including the difficulty of preventing water containing essential oil from entering certain cages. Two different populations (flocks) reared under the same conditions were observed and monitored before and after the administration of marketed OEO for main production parameters, involving the entire population (hens) present in each flock, while blood samples were collected for biochemical analysis from randomly selected subsamples. The pre-treatment period was considered as a baseline or initial reference point to measure changes after OEO supplementation. It was carried out for three weeks, from the beginning of April 2024 (spring), on two flocks belonging to two separate houses of a commercial poultry farm located in Constantine province (Algeria) owned by the same poultry producer and under the same management conditions. This study was conducted on 7884 Hy-Line Brown late-phase laying hens at different ages depending on the flock. The flocks were recruited for their production and health record. The first flock (flock 1), which comprised the oldest hens (*n* = 3849 96-week-old hens), exhibited a normal production curve with a peak laying rate of 93.60% at 32 weeks of age (Figure 1). In comparison, flock 2, consisting of the youngest hens (*n* = 4035 79-week-old hens), had an abnormal production curve, where production reached its maximum rate of 69.32% at 37 weeks and displayed a period of production decline reaching 46.53% at 44 weeks (Figure 1). Both flocks also experienced certain periods of high mortality, reaching a maximum weekly mortality of 0.40% and 0.47% in flock 1 and flock 2, respectively. However, the frequency and magnitude of mortality were different between the two flocks. They were more pronounced with higher peaks in flock 2 (Figure 1). These problems led the farmer to use antibiotics (erythromycin and tetracycline) as well as vitamins (AD3E) frequently during laying phases. No therapeutic molecules were administered to any hens for at least 10 weeks before the start of the experiment.

The birds were received at the farm as pullets, after having undergone a different rearing period (1 week to 16 weeks of age) for each flock, and began the laying period from the age of 18 weeks. All hens were reared under the same conventional cage system of production (43 cm × 42 cm × 41 cm; four birds per cage) and the same breeding conditions, namely, light, temperature, and diet. The layer houses had controlled ventilation with a temperature maintained at approximately 24 ± 1 °C and a photoperiod of 15 h daily “(5W LED)” during the experimental period. The birds received water ad libitum and had free access to feed, manually distributed at 8 a.m. (Table 1), according to the Hy-Line Brown management guide [32]. The poultry farmer decided to end the production cycle of both flocks during the fourth week after the start of the OEO administration (at the ages of 99 and 82 weeks for flocks 1 and 2, respectively) for economic (market price) and zootechnical reasons in order to practice a sanitary vacuum before the installation of new flocks.

### 2.2. Oregano Essential Oil Administration

**Figure 2:**
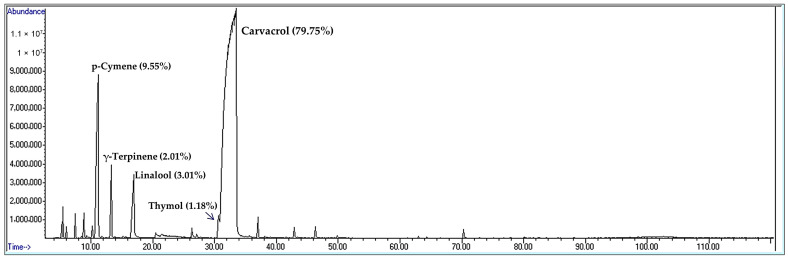
Main phytochemical composition of studied commercial oregano essential oil (*Origanum heracleoticum*).

The oregano essential oil (OEO) used was a phytogenic eubiotic commercial natural product containing 5% of *Origanum heracleoticum* essential oil. Its main ingredient was 5% active compounds consisting mainly of carvacrol (79.75%), as presented in Figure 2, and 95% of an inactive carrier. The phytochemical composition of OEO was carried out in the Scientific and Technical Research Center in Physico-Chemical Analyses (CRAPC, Bou Ismaïl, Tipaza, Algeria) by Gas Chromatography–Mass Spectrometry (GC-MS) using an Agilent GC 6890 plus gas chromatograph coupled to an Agilent 5973 quadrupole mass spectrometer (Agilent Technologies, Santa Clara, CA, USA), equipped with an apolar HP-5MS capillary column (5% phenyl, 95% dimethylpolysiloxane, 30 m × 0.25 mm × 0.25 μm film thickness). Based on the manufacturer-prescribed dosage, the farmer administered the commercial OEO on the same day for both flocks for 7 days, from week 96 for flock 1 and from week 79 for flock 2, via drinking water at a maximum inclusion rate of 300 mL of product per 1000 L of drinking water, corresponding to 14 mg of pure OEO per liter of water, for a density of 0.93 g/mL of essential oil, without any withdrawal period.

### 2.3. Performance Production Metrics

**Figure 3:**
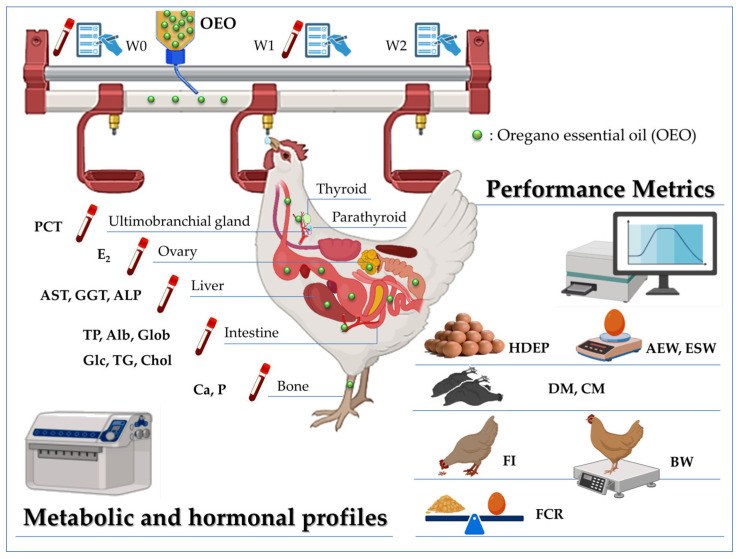
Study protocol applied to the two studied flocks during different treatment periods. Oregano essential oil (OEO), week (W), procalcitonin (PCT), estradiol (E_2_), total protein (TP); albumin (Alb), total globulin (Glob), glucose (Glc), triglycerides, total cholesterol (TC), aspartate aminotransferase (AST), gamma glutamyl transferase (GGT), alkaline phosphatase (ALP), calcium (Ca), phosphorus (P), hen-day egg production (HDEP), average egg weight (AEM), eggshell weight (ESW), daily mortality (DM), cumulative mortality (CM), feed intake (FI), body weight (BW), and feed conversion ratio (FCR).

Data for all present late-phase laying hens per flock (*n* = 3849 for flock 1 and *n* = 4035 for flock 2) were collected from daily flock records. The number of eggs (normal and broken) and dead birds was recorded daily by the farmer, according to their usual flock management schedule. The provided daily feed was weighed, and the feed retained was appreciated at the end of the day to calculate daily feed consumption. The performance of each flock was calculated once before the treatment at week 0 (Pre-OEO Tx), at the end of the treatment at week 1 (OEO Txt-Week), and one week after the end of the treatment at week 2 (Post-OEO Tx Week). In addition to the recorded indices described above, the following flock performance parameters (Figure 3) were estimated for each treatment-related period, except for FI, FCR, and BW, calculated only before and at the end of the treatment week using formulas inspired by the Hy-Line Brown management guide [32]. The obtained performances were compared with standard values retrieved from this guide. The average egg weight (AEW) used to calculate the FCR was estimated daily on a random sample of 300 eggs. To assess the quality of the eggs, mainly the weight of the shell (ESW) and eggshell percentage [33], 10 eggs per treatment period (5 eggs per flock) were randomly sampled and sent to a state-approved laboratory for food quality control evaluation. The AEW reported in the Results and Discussion Sections are those obtained from the laboratory evaluation to highlight exactly its relationship with ESW.

Percent Hen-Day Egg Production (HDEP) (%) = (Number of eggs produced in one day)/(Current hen inventory)

Daily Mortality = (Total dead birds for the day)/(birds day)

Cumulative Mortality = (Total dead birds to date)/(birds housed)

Average Egg Weight (AEW) and Eggshell Weight (ESW) in g = Average egg and its shell weight of every week by randomly collecting 10 eggs per treatment period (5 eggs per flock)

Eggshell Percentage (%) = (Average Eggshell Mass/Average Egg Weight) × 100

Feed Conversion Rate (FCR) = kg of feed consumed during the period/kg of egg mass produced during the period (Number of eggs × Average Egg Weight of a random sample of 300 eggs)

Average Body Weight (kg) = Average bird weight of 10 randomly selected birds per flock with 5 birds sample for each replicate

### 2.4. Metabolic and Hormonal Profiles

A weekly metabolite and endocrine profiling in blood plasma was established before the administration of commercial oregano essential oil on day 0 (Pre-OEO Tx) and 7 days later at the end of the treatment period (Post-OEO Tx).

A total of 40 blood samples were collected at 8 a.m. before feed delivery by venipuncture from the brachial (wing) vein and placed in encoded heparinized tubes (3 mL per Bird) from two subsamples of 5 randomly selected laying hens from each flock, forming a group of a total of 10 hens per flock at each treatment period (*n* = 10 birds). These hens were considered as individual biological units obtained, for both time points, from the same cage/location and under identical rearing conditions, according to Hy-Line International recommendations [34]. The plasma was then separated by centrifugation at 3000 rpm for 10 min, which was kept under short-term (a few hours) storage conditions (4–8 °C) for biochemical analyses and stored at −20 °C until hormonal analyses.

Plasma concentrations of common biochemical markers of protein nutrition (total protein, albumin, and total globulin), energetic status (glucose, triglycerides, and total cholesterol), liver function (aspartate aminotransferase, gamma glutamyl transferase, and alkaline phosphatase), bone and mineral metabolism (calcium, phosphorus) were determined by an automated chemistry analyzer (Cobas Integra 400 Plus) using commercially kits (Roche Diagnostics International Ltd., CH-6343 Rotkreuz, Basel, Switzerland). Moreover, the albumin/globulin and calcium/phosphorus ratios were then calculated.

For the endocrine profile, ovarian function was explored by assessing the estradiol (E_2_) plasma concentration by an automated analyzer (Maglumi X3) using a Chemiluminescence Immunoassay (CLIA) via a commercial kit (Snipe Diagnostic, Shenzhen New Industry Biomedical Engineering Co., Ltd., Shenzhen, China). For bacterial infection and inflammation diagnosis, the concentration of procalcitonin (PCT) was measured in the hen plasma by a CLIA System (Mindray CL-2000i) using a commercial kit (Shenzhen Mindray Bio-Medical Electronics Co., Ltd., Shenzhen, China) (Figure 3).

### 2.5. Statistical Analysis

Data on the daily performance production and those of the studied biomarkers measured in the two flocks during different periods of treatment were statistically analyzed using IBM^®^ SPSS^®^ Statistics 26 software. The normal distribution of data, using the Shapiro–Wilk test, and homogeneity of variance, using Levene’s test, were verified before being subjected to variance analysis. This study included two independent populations: flock 1 and flock 2. Within each flock, the pre- and post-treatment measurements were considered as dependent samples when they were obtained from all present hens. The main parameters, i.e., HDEP, CM, and DM, calculated on all present birds before OEO administration (W0), at the end of treatment (W1), and one week after treatment period (W2) were compared within the flocks using repeated-measures ANOVA with the Least Significant Difference (LSD) test. The Friedman test was used instead of repeated-measures ANOVA when the normality assumption was violated. For the FCR, the paired *t*-test was used to determine if there was a significant difference between the means before and after treatment. However, instead of this *t*-test, the related-samples Wilcoxon Signed Rank test was applied for this parameter and for FI when the normality assumption was violated. For egg quality and live weight, the comparison of means between independent groups, randomly formed and defined by treatment-related periods, was performed using ANOVA with an LSD post hoc test to compare each pair of means and *t*-tests for two independent groups (before and after), respectively. To assess the effect of oregano essential oil, the variance in the studied biochemical parameters before (Pre-Tx) and after OEO administration (Post-Tx) was analyzed in the two laying hen flocks. A standard general model analysis (GLM) with fixed effects was conducted, considering flock (two independent populations with different origins, histories, and ages) and treatment (pre-treatment and post-treatment periods) as fixed effects. The model tested the main effects and the “flock × treatment” interaction. This interaction tested whether changes from before to after OEO administration differed between the flocks. The model did not include a repeated-measures structure or a subject-related random effect, as different individuals were sampled at each time point. Baseline (Pre-Tx) and follow-up (Post-Tx) mean values of metabolic and hormonal parameters (dependent variables) were compared using the Bonferroni post hoc test. Furthermore, to estimate the relationship between endocrine (PCT and E_2_) and metabolic markers (TP, Alb, Glob, Alb/Glob, Glc, TG, Chol, AST, GGT, ALP, Ca, P, and Ca/P), the Pearson correlation was executed. For all tests, a significance level of 0.05 (5%) was set.

## 3. Results

### 3.1. Effect of Oregano Essential Oil on Flock Performance Metrics

#### 3.1.1. Egg Production and Egg Quality

**Figure 4:**
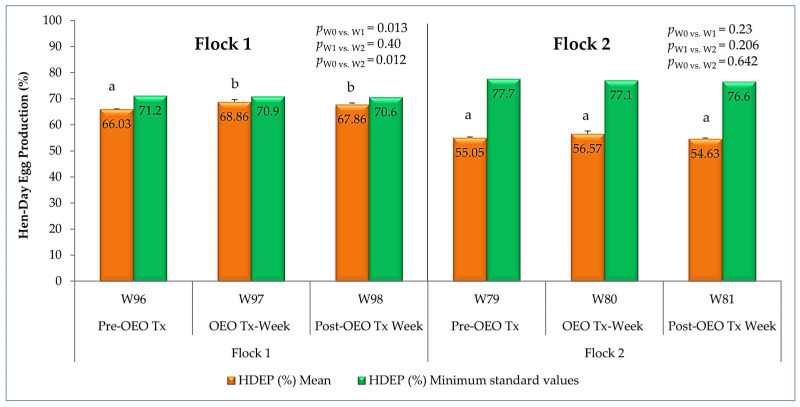
Variation in hen-day egg production (HDEP) means by flock during different OEO treatment periods. For each flock, bars with different letters (a, b) for treatment-related periods are significantly different at *p* < 0.05 using the LSD test.

The impact of oregano essential oil administration on the egg production of late-phase laying hens per flock is shown in Figure 4. For flock 1, including the oldest late-phase laying hens, the hen-day egg production (HDEP) showed a significant improvement (*p* < 0.05) during treatment, increasing from the pre-treatment to the end of the treatment week, while maintaining a high level of 67.86%, close to Hy-Line Brown guideline values, one week after the cessation of treatment. The youngest late-phase laying hens in flock 2 showed a similar enhancement in HDEP but less pronounced results compared to the standard values (Figure 4).

#### 3.1.2. Egg Quality

The average egg weight, eggshell weight, and shell percentage showed different variations depending on the flock (Table 2). Aged hens (Flock 1) showed a slight, non-significant improvement (*p* > 0.05) in average egg weight at the end of the treatment before stabilizing at around 68.44 g one week after the treatment was stopped, while maintaining values that slightly exceeded those of the breed guide. In contrast, the younger hens in flock 2 showed a significant improvement (*p* < 0.05) in egg weight during treatment, rising from a value (61.66 g) below the standard to a weight of 71.38 g, representing a marked increase of 9.72 g (15.76%). This increase did not last a week after the treatment ended, decreasing but still exceeding the standard value of 65 g.

Contrary to flock 1, which did not show a significant increase in shell weight (*p* > 0.05), a significant improvement was observed in flock 2, where shell weight reached 10.04 g one week after the cessation of treatment (*p* < 0.05). In this flock, among the youngest hens, shell percentage decreased significantly (*p* < 0.05) at the end of treatment to 11.51%, where it approached the maximum reference value of 11%. Excluding this effect, the percentages recorded in both groups and in the different trial periods were higher than the reference value described above (Table 2).

#### 3.1.3. Egg Production Efficiency, Growth, and Development

**Figure 5:**
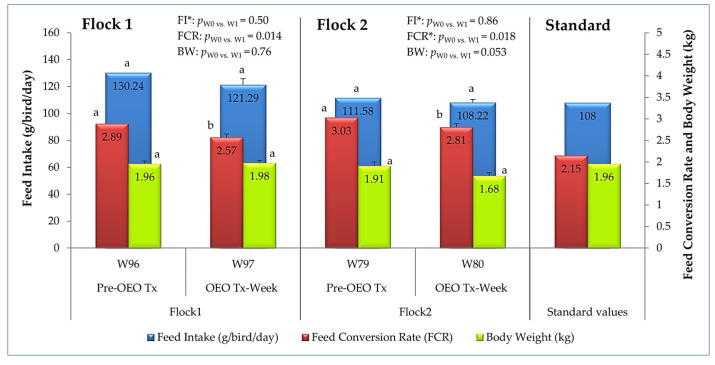
Means of feed intake, feed conversion rate, and body weight by flock before and after OEO administration. For FCR in flock 1, mean bars with different letters (a, b) are significantly different at *p* < 0.05 using a paired *t*-test. *: For FCR in flock 2 and for FI in each flock, mean bars with different letters (a, b) are significantly different at *p* < 0.05 using the Wilcoxon Signed Rank test. Body weight means were compared using a *t*-test with a significance level of 0.05.

After one week of OEO oral administration, a non-significant decrease (*p* > 0.05) in feed intake was recorded in both studied flocks, although the decrease was more pronounced in older hens (Flock 1), with values far from the standard. Aromatherapy significantly influenced (*p* < 0.05) feed efficiency by reducing the feed conversion rates calculated in both flocks. For flock 1, the FCR improved significantly from 2.89 to 2.57 during treatment. However, its values did not meet the breed standard among the study periods (Figure 5).

The addition of OEO did not have a significant effect (*p* > 0.05) on body weight. Only flock 2 showed a slight decrease in live weight. It had, from the beginning, unlike flock 1, a lower than standard weight (Figure 5).

#### 3.1.4. Livability (Mortality)

**Figure 6:**
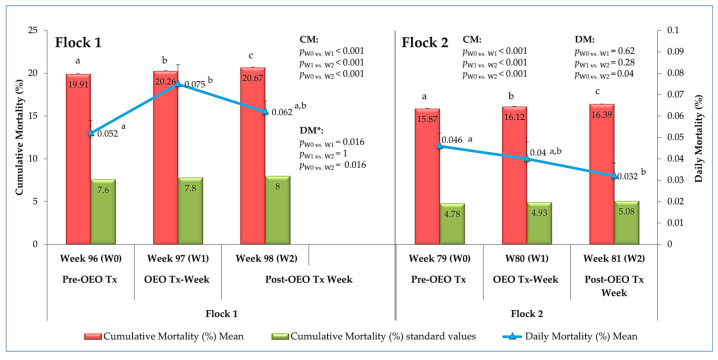
Changes in cumulative mortality and daily mortality by flock during different OEO treatment-related periods. For each flock and for each parameter, mean bars with different letters (a, b, c) are significantly different at *p* < 0.05 using the “LSD” test. * Daily mortality means in flock 1 are significantly different at *p* < 0.05 using the Friedman test.

Before receiving OEO, the two flocks showed very high cumulative mortality rates compared to the Hy-Line Brown variety standard (19.91% vs. 7.60% for flock 1 and 15.87% vs. 4.78% for flock 2). At the end of treatment and one week later, a significant evolution (*p* < 0.05) in this parameter was observed in both flocks. This increase, estimated at an average of 0.38% and 0.26% per week in flock 1 and flock 2, respectively, exceeds the acceptable limits of 0.2% per week variation described in the management guide, notably in the aged layers in flock 2. In contrast to the significant increase observed in the older hens in flock 1, a significant decrease in daily mortality was observed (*p* < 0.05) in flock 2 during treatment and one week after the end of treatment (Figure 6).

### 3.2. Effect of Oregano Essential Oil on Metabolic Profile

The blood biochemical parameters of the hens from the two flocks before and after administering commercial oregano essential oil in drinking water are shown in Table 3.

#### 3.2.1. Protein Nutrition

The factorial analysis of changes in protein plasma levels (total protein, globulins, albumin, and albumin/globulin ratio) reveals the only significant main effect of supplementing drinking water with oregano essential oil (*p* < 0.05), without dependence on the age and rearing conditions of the hens (flock effect) or their interaction with the treatment (*p* > 0.05). Although total protein and globulin remained within the reference range before and after the addition of OEO, this aromatherapy significantly increased the plasma concentration of these two parameters (*p* < 0.01). However, the plasma albumin concentration, which was below the reference values before treatment, continued to decrease significantly during the trial (*p* < 0.05), in parallel with a significant reduction in the albumin/globulin ratio under the threshold value after treatment (Table 3).

#### 3.2.2. Energy Status (Carbohydrate and Lipid)

Energy status markers, namely, glucose (carbohydrate), total cholesterol, and triglycerides (lipids), were not influenced by any factor, neither the flock (*p* > 0.05), the OEO addition (*p* > 0.05), nor their interaction (*p* > 0.05). Plasma values for these parameters, before and after treatment, were within the reference ranges despite insignificant decreases one week after receiving OEO (Table 3).

#### 3.2.3. Liver Function

The exploration of liver function by measuring the AST, GGT, and ALP enzymes in the blood of the laying hens in both flocks showed a significant effect of OEO treatment on AST concentrations only (*p* < 0.05). These concentrations decreased significantly by approximately 60 IU after this oral therapy, although these variations were within the reference ranges. However, plasma GGT concentrations were not affected by treatment (*p* > 0.05) despite normalization of their values after treatment, and ALP concentrations were within normal limits, regardless of the flock of origin and age, as well as treatment (*p* > 0.05) over the course of the trial (Table 3).

#### 3.2.4. Mineral Metabolism

The analysis of variations in calcium–phosphorus balance (Table 3) showed only a significant effect (*p* < 0.05) of OEO supplementation in water on the three parameters measured (Ca, P, and Ca/P), regardless of the flock (*p* > 0.05). Plasma Ca levels and, in parallel, the Ca/P ratio, which exceeded the thresholds before treatment, returned to normal with significant reductions in their values at the end of treatment (*p* < 0.05 and *p* < 0.01, respectively). Unlike this, plasma phosphorus concentrations remained within normal limits throughout the trial and increased significantly (*p* < 0.05) at the end of OEO administration, resulting in a substantially balanced Ca/P ratio after treatment (Table 3).

### 3.3. Effect of OEO on Endocrine Profile

Variations in the plasma concentrations of procalcitonin, a biomarker of inflammation and bacterial infection, in the samples from both flocks are significant and only influenced by the administration of OEO (*p* < 0.05) without intervention or interaction with the flock effect (*p* > 0.05). They decreased significantly (*p* = 0.01), with a 55% reduction corresponding to 75 pg/mL after one week of treatment. The kinetics of procalcitonin were significantly but mildly positively correlated with liver enzymes, more so with GGT (*p* < 0.01) than with AST (*p* < 0.05) (Table 4). The plasma levels of the ovarian steroid hormone, estradiol, were significantly affected by the addition of OEO (*p* = 0.01) and independently of other factors of variation included in this study. Their values increased significantly by approximately 510 pg/mL after treatment in both flocks (Table 4). Their kinetics were significantly in a weak negative relationship with those of albumin (*p* < 0.05) and the albumin/globulin ratio (*p* < 0.01), showing an intermediate level of correlation with AST and the Ca/P ratio (*p* < 0.01) (Table 4).

## 4. Discussion

This study was conducted using a before–after design without a control group, combining several zootechnical and biochemical parameters with the innovative introduction of procalcitonin as a biomarker of bacterial infection and inflammatory status in laying hens. However, due to real-world conditions, methodological limitations, including statistical restrictions related to sample size, the results obtained allow the formulation of hypotheses in the form of associative trends rather than direct causal findings regarding the effects of OEO supplementation.

The effects of oregano essential oil (OEO) administered in drinking water vary depending on the flock (age and medical and rearing histories of the hens), with improved production performance observed in older-elderly hens (flock 1), whose performance was close to standard before treatment. However, changes in egg quality and shell quality were observed in younger-elderly hens (flock 2), which had low production and lower egg quality compared to standard values prior to this water supplementation.

Oregano essential oil demonstrated a significant positive effect on egg production, particularly noticeable in older hens (96 to 98 weeks) in flock 1, with an increase of almost 3% during treatment and a sustained effect, as this improvement was maintained one week later. This is consistent with the benefits of supplementing hen feed with OEO reported in recent studies confirming its effectiveness in egg production, although this impact varies depending on experimental conditions [15,16]. The improvement in hen-day egg production (HDEP) can be explained by the fact that oregano essential oil contains phenolic compounds (mainly carvacrol in this study) with antimicrobial and anti-inflammatory properties [10,37] that can improve digestive health, including intestinal morphology and structure in late-phase laying hens [17,19] and the immune status of hens [37,38]. As a result, improved nutrient absorption directly translates into an increased ability to form and lay eggs [15]. The fact that this effect persists for a week after the treatment period suggests a beneficial remodeling of the intestinal microbiota. However, some research reports that supplementation with OEO has no significant influence on egg production during the peak laying phase of Hy-Line Brown layers housed in an environmentally controlled house [25] or during the first few weeks following the addition of *Origanum syriacum* L. essential oil in Lohman hens [20]. There was also no impact when this oil was administered in combination with other oils [39], suggesting that the effect of OEO on laying rate varies depending on the age of the hens, the duration of treatment, whether it is administered alone or in combination, its composition, including the primary bioactive compound, and the strain or breed of hen [14]. The efficacy of oregano essential oil seems to depend on its phenolic compound concentration. The present dosage (14 mg of pure OEO/L of water) ensures a dose of 2.28–3.25 mg of OEO/bird/day, considering a standard water intake of 163–232 mL/bird/day during the late production phase, as mentioned in the Hy-Line hens guide [32]. This is in accordance with that of 25 mg/kg used by Xian et al. [19], corresponding to 2.7–2.9 mg of OEO/bird/day when considering a standard feed intake of 108–116 g/bird/day, and that of 24 mg/kg tested by Bozkurt et al. [40], corresponding to 2.59–2.78 mg of OEO/bird/day, considering a standard feed intake of 108–116 g/bird/day. The present dosage ensures a dose of 1.82–2.59 mg of carvacrol/bird/day, which is similar to or exceeds the dosage of 1.98–2.12 mg of carvacrol/bird/day obtained from the high concentration of 200 mg of OEO (containing 9.18% carvacrol) per kg of diet corresponding to 21.6–23.2 mg of OEO/bird/day evaluated by Migliorini et al. [38]. The variation in findings is well illustrated by the results obtained at the flock 2 level, which shows no significant improvement and maintains low values of HDEP compared to standard values [32], suggesting variability in the response to treatment depending on the rearing conditions or the age of the hens. However, in this flock comprising the younger-old hens, a significant improvement in egg weight was observed. Egg mass was lower than the standard value for Hy-Line Brown hens [32] prior to treatment, with an increase of nearly 10 g at the end of the treatment period, in line with the results of other authors [16,25,39]. The significant decrease in the eggshell percentage approached but remained above the reference value [33]. Although this represents a relative reduction in shell proportion alongside an increase in egg mass, the absolute shell weight increased after treatment, indicating that shell-forming capacity was maintained despite larger egg size. This is consistent with the results of other studies on stored eggs from hens supplemented with 50 mg OEO/kg [41] but not with those of other authors who did not observe a significant effect when they used commercial oregano essential oil in the same strain (Hy-Line Brown) during peak production [25]. Furthermore, the decrease in the shell ratio is not supported by studies that tested oregano and thyme in herb form [42] or by results indicating a decrease in egg mass after adding OEO (*Origanum vulgare* L.) to the basal diet of Lohmann LSL-Lite laying hens at 66 weeks of age for 12 weeks [43]. Similarly, an effect on eggshell weight after treatment, indicating a delayed but positive effect on shell strength, was recorded, in line with the observations of others [20]. This was also observed in the Isa Brown breed following the addition of Mexican oregano essential oil from a different genus, *Lippia origanoides*, which does not belong to the Lamiaceae family but is also known for its high carvacrol and thymol content [44]. Conversely, no significant effect on egg weight was found in young Lohmann laying hens (32 weeks old) receiving dried oregano (*Origanum vulgare* subsp. *Hirtum*) mixed with rosemary [45]. Furthermore, the administration of oregano essential oil mixed with other oils did not affect eggshell weight [16,39]. Variations in egg weight, shell weight, and shell percentage can provide information about the calcium metabolism of hens. In flock 2, a late improvement in shell weight (one week after treatment cessation) with, inversely, a temporary decrease in shell percentage during treatment, was observed. This contradiction can probably be explained by a temporal dynamic, i.e., oregano oil may first promote whole egg growth (hence, the relative decrease in shell weight) and then secondarily improve calcium mineralization [17,41,44]. Another explanation may be the beneficial effect of adding oregano essential oil on egg quality via gut microbiota modification in laying hens at 25 mg/kg of dietary oregano oil [19]. However, due to the size of the egg sample, the results relating to their quality may be of secondary interest, serving more as an associative observation than a conclusive finding on the effects of OEO.

Regardless of the flock of origin and the age of the hens, and in accordance with some studies, supplementation with oregano essential oil has no significant influence on the average daily feed intake of laying hens [25,45,46,47], nor that of pullets [48]. Nevertheless, a downward trend in feed consumption during treatment is to be emphasized, suggesting an optimization of feed efficiency. This efficiency is well illustrated by the significant improvement in feed conversion rates in both flocks, resulting in a considerable economic advantage. Thus, by reducing the FCR, oregano essential oil can enable the production of more kilograms of eggs with fewer kilograms of feed. These results are consistent with those reported by several authors [15,25,41,46]. This is probably because oregano essential oil, which is rich in carvacrol, as in this experiment, and thymol [10], can reduce pathogenic bacteria while promoting beneficial bacteria, thereby improving digestion, modifying intestinal structure and morphology, and resulting in more efficient nutrient absorption and optimized feed conversion [15,19,37,48]. The results of FCR improvement cited in the literature were dependent on the conditions of the experiment, particularly the dose of essential oil. For example, during winter, productive efficiency can improve even at doses of 39.8 mg of oregano essential oil per kg when considering feed conversion [41]. Other authors, instead, found no impact of OEO on feed conversion [20,45,47] or an increase in its value after treatment [43]. A meta-analysis study including several essential oils added to hen feed concluded that these additives could have an effect on egg production and quality, as well as feed conversion, according to hen age and the main bioactive compound, but not depending on the breed or strain, the supplementation period, or the dose of essential oils [14].

The insignificance of changes in body weight after administration of OEO in both flocks has been noted in other studies [47]. This could be explained by the lack of impact of this oil on dietary intake, as noted above. The weight loss recorded was probably related to the slight decrease in consumption. Moreover, this marked loss, which was more pronounced in the second flock of younger-elderly birds, probably indicates a metabolic adaptation through the mobilization of body fat reserves to ensure the remarkable 10% increase in egg weight at the end of the treatment. It has been suggested that the consumption of dietary added fats may increase egg productivity and weight by stimulating the synthesis of oviduct proteins, which are essential for egg formation, through the action of estrogen [49]. Nevertheless, the benefits of oregano essential oil supplementation reported in the literature include increased body weight gain, although results may vary depending on age, especially for growing birds such as pullets [48], and experimental conditions [44].

Few studies have involved mortality to monitor the effect of essential oils on the performance of laying hens. In the present study, cumulative mortality before the trial greatly exceeded the standards for the breed in both flocks [32]. During treatment and one week after, a statistically significant increase in cumulative mortality was recorded in both flocks, but with a variation greater than the standard value of 0.2%, particularly in older hens (flock 1), which probably indicates a decline in survival as hens age [50,51]. A significant but inconsistent increase in daily mortality in flock 1 was recorded in aged hens undergoing natural decline (aging). Thus, its significant decrease in flock 2, unlike flock 1, may indicate differential responses reflecting the interaction between age-related physiological decline and pre-existing health status, or there may be statistical reasons related to the normality of the data distribution rather than the effects of the treatment alone. Other studies reported no significant difference in the mortality rate observed during the experimental period between treated and control groups [46]. It should be noted that the parameter “mortality” was discussed in several studies conducted under commercial farming conditions because, under these conditions, mortality is multifactorial, such as infectious disease, housing systems, and variations between breeds [50,51,52], and cannot be simply attributed to the use, posology, duration, or main compound of the treatment.

Therefore, the divergence in the findings regarding the post-treatment production performances requires further analysis of the observed effects, considering the results of metabolic and hormonal profiles established in this study.

The biochemical profile provides information on metabolic changes [53], nutritional status, and its impact on liver function through the assessment of enzyme levels [54] and potential disorders, helping in the diagnosis of diseases and ensuring optimal management practices and production performance [55]. It can be an indicator of the impact of intrinsic factors, such as the production phase or age of hens, or extrinsic factors, such as housing conditions [56].

Plasma protein profiling shows that concentrations of total protein (TP) and globulin increased significantly and only under the effect of the addition of oregano essential oil, but still within the reference range. These elevations are similar to those observed in hens older than 59 weeks after 28 days of feeding OEO at 150 mg and 200 mg per kg of diet, leading the authors to suggest that OEO has an effect on the anti-inflammatory response by elevating immunoglobulin levels [38]. Even at a low inclusion level, adding 20 g/kg of oregano herb maximized the TP concentration in 60-week-old Hy-Line Brown laying hens [42]. Total protein levels reflect the cumulative values of plasma proteins, including albumin and globulin, providing a general overview of protein homeostasis through their colloidal (oncotic) osmotic pressure [57]. The present elevation in TP concentrations may be related to the elevation in the level of its component, globulin, observed in this study. Globulins are synthesized in several forms (alpha, beta, and gamma) in chickens depending on several factors, including age, production cycle, diet, and farming practices, as well as the environment [57,58]. Gamma globulins comprise non-immunoglobulin fractions and immunoglobulins (Igs). Non-immunoglobulins (transferrin family) are synthesized by the liver and oviduct and released into the blood [57]. Ovotransferrin, present in both plasma and eggs, is the only soluble glycoprotein of the transferrin family in chickens. Ovotransferrins, synthesized by the liver (hepatocytes) and secreted into the blood, are influenced by pro-inflammatory cytokines, whereas estrogens control ovotransferrin production, which is incorporated into the egg at the oviduct [59]. Their increases may indicate an improvement in liver and oviduct function in response to the protective antioxidant, anti-inflammatory, and anti-infectious effects of phenolic compounds, particularly carvacrol [10,37], on secretory cells. Immunoglobulin fractions are components of the immune system (IgY, IgA, IgM) produced in adult hens by B lymphocytes in secondary lymphoid organs, such as the spleen, bone marrow, and lymphoid follicles of the oviduct [59]. In laying hens, the oviduct is metabolically very active, enabling the transfer of immunoglobulins to the egg yolk, thus explaining the nutritional quality of this animal product. The increase in immunoglobulins [38], as a gamma globulin component, is probably related to a possible stimulation of this immune function following the addition of OEO, indicating an anti-inflammatory response, an improvement in hen health, and a response to an alteration in the digestive bacterial flora. However, in this study, only total globulin was measured without evaluating its specific components (such as immunoglobulins). Therefore, the hypothesis formulated on the possible relationship between the variation in globulin levels and stimulation of immunity in response to OEO administration suggests that further studies on specific immunoglobulins are needed before definitive findings can be drawn. Metabolically speaking, an increase in globulin levels means improved digestive efficiency and better absorption of amino acids derived from the digestion of ingested proteins in the intestine, confirming the beneficial effect of OEO on intestinal structure, function, and health, including the cecal flora, as reported by several authors [14,17,20]. This can be explained by improved absorption of excess dietary protein, given that the crude protein content of the feed distributed to both flocks (19%) exceeded the recommendations of 14.09% and 12.92% CP for feed intakes of 110 g and 120 g/bird/day, respectively, as described in the breeding guide [32]. In contrast, other authors [40] did not find results consistent with those of the present study; rather, they found a non-significant reduction in TP levels in Lohmann White strain laying hens between 82 and 106 weeks of age fed a standard diet supplemented with OEO. Albumin is another fraction of proteins that are mainly involved in the transport of lipophilic hormones, including steroid hormones in plasma, and the synthesis of egg yolk, in particular [60,61]. It was already below normal levels before the experiment, and its levels showed a significant decrease after the addition of OEO, resulting in a decrease in the albumin/globulin ratio. This decrease is similar to that observed in a previous study on hens after 84 days of supplementation with OEO at a high dose of 200 mg/kg [38]. Furthermore, the difference in kinetics between globulin and albumin can be attributed to the fact that sex steroid-binding globulin is not present in birds and that albumin binds to lipids, including fatty acids, transports, and distributes them [57]. Although albumin serves as a transport protein for lipophilic hormones, particularly estradiol, and contributes to the formation of egg yolk proteins, the mechanisms underlying this inverse relationship remain unclear. Further research, including analysis of hepatic gene expression, would be needed to elucidate these mechanisms. These latter effects appear to be associated with a possible stimulating effect of OEO on reproductive function, mainly egg-laying, as indicated above by the egg production rate (HDEP) and follicular growth, denoted below by the estrogen level.

The plasma concentrations of glucose, total cholesterol, and triglycerides in this study were not influenced by any factors, including the addition of OEO to drinking water for 7 days. They remained within the reference range [35] before and after administration of the essential oil, indicating that the ration distributed, which provides 2734 kcal of metabolizable energy per kg of feed, adequately covers the energy requirements of the hens. This energy value ensures variation ranges of 328 kcal and 296 kcal/bird/day for daily feed intakes varying between 120 g and 108 g/bird/day after treatment in flocks 1 and 2, respectively. This is close to the recommendations of the 300–315 kcal/bird/day described in the Hy-Line Brown breeding manual for hens in the fifth and final phase of their laying cycle, with production below 83% and consuming between 100 g and 120 g/bird/day daily [32]. The decrease in daily feed intake and metabolizable energy at the end of treatment explains the normal, non-significant decrease in these parameters at the end of the trial. The lack of an OEO effect has also been reported in other studies for glucose [39,42], triglyceride, and cholesterol [38,39,48] levels. However, this natural additive has been shown to reduce triglyceride levels in pullets [48] and young laying hens [25], as well as on cholesterol concentrations at the start of laying [25] or during the fourth phase of production (60 weeks of age) in Hy-Line Brown hens receiving 20 g of oregano herb/kg of feed providing 2766 kcal/kg of metabolizable energy [42]. Conversely, a cholesterol-increasing effect has been reported in pullets at 11 weeks of age [48] and in laying hens at the end of their productive life (over 82 weeks), along with a moderate hyperglycemic effect within normal limits [40]. Birds exhibit naturally increased blood glucose levels and rely on glucagon, mainly, and alternative metabolic adaptations to regulate energy balance, such as an enhancement in antioxidant systems that reduce the oxidative stress responsible for cellular damage [62]. This suggests that OEO has a reinforcing effect, thanks to its proven antioxidant properties, on the chicken’s innate antioxidant system, leading to a decrease in blood glucose levels while keeping them within normal ranges, so that hens can adapt to the stress associated with increased egg production. Nevertheless, in the literature, variations in the levels of these parameters have been attributed to several physiological factors, including age and nutrition [57], and environmental factors, such as heat stress [22]. They have also been linked to pathological factors like liver damage, such as steatosis in laying hens [35] or lesions found in the organs of 95-week-old laying hens [63].

The parameters discussed above (protein and energy) clearly show the crucial role of the liver in laying hens in homeostasis and in lipid and glucose metabolism for the formation of egg yolk. They also highlight the liver’s involvement in protein metabolism for the formation of egg yolk (albumin) and immunity (immunoglobulin). The liver also participates in calcium metabolism (shell formation) and reproduction via estrogen receptors that control the synthesis of egg yolk precursors, namely, vitellogenin (VTG II) and apolipoprotein (VLDL II), and the synthesis of growth factors [57,64,65]. This key organ is generally critical to health due to its roles in antioxidant stress and detoxification processes [66,67]. These findings justify the importance of investigating liver function integrity after administration of OEO. It has been suggested that measuring the liver enzymes AST, GGT, and ALP, although not specific to liver damage due to their physiological and environmental variations [57], can assist in verifying the effectiveness of food additives, such as medicinal plant extracts [23] or their possible dose-dependent disruptive effect on liver metabolism [68]. Liver function can then be assessed using blood levels of AST and GGT, for the integrity of hepatocytes and their membranes, and ALP levels, for bile excretion [35]. In this study, all enzymes explored showed a decrease in their levels after treatment, suggesting the safety of the dose applied for all birds. The decrease in GGT, a marker of cholestasis, hepatitis, and steatosis [35], was not significant, as noted in a previous study, but with a mixture of essential oils containing thymol [68]. However, GGT levels exceeded normal ranges prior to treatment in younger hens from flock 2 with a poor medical history (low production and moderate livability), which probably indicates chronic hepatobiliary impairment in these hens as well as subclinical liver damage or stress. Some authors have attributed the increase in GGT and AST in laying hens to high nutritional demand during peak laying periods [54]. However, in the present study conducted on hens at the end of their laying career, only the decrease in plasma AST was significantly influenced by the addition of OEO to the hens’ drinking water, regardless of the hens’ age and flock of origin. This decrease was similar to that recorded following the addition of dietary oregano essential oil but in young birds, such as pullets [48] and laying hens at 36 weeks of age [25]. Contrary to this, the latter authors [25] reported an increase in serum AST levels at 40 weeks, suggesting variations in the activity of this enzyme depending on age. Recent studies suggest that age may cause impaired liver function in laying hens through increased lipid peroxidation and protein oxidation, leading to delayed production and decreased liver health with advancing age in laying hens [69,70,71]. This suggests that decreases in liver enzyme activity in elderly (over 79 weeks) or very elderly hens (over 96 weeks), significant for AST and not significant for GGT and ALP, could be markers of increased resistance to hepatocyte oxidative stress provided by OEO via the antioxidant and anti-inflammatory cell-protective properties of carvacrol described in the literature [10,37,72]. Moreover, the normalization of GGT values post-treatment in flock 2, though not statistically significant, supports the hepatoprotective hypothesis against possible subclinical liver damage or stress. However, distinguishing between active hepatoprotection versus cessation of hepatotoxic stress requires controlled studies with histopathological examination and hepatic oxidative stress markers. In the present study, the measurement of circulating antioxidant enzymes, including superoxide dismutase, catalase, and glutathione peroxidase, could have made the exploration of the effect of OEO on the antioxidant capacity of the liver in these aged hens more complete and specific. The absence of a significant effect on ALP concentrations in this study, which is similar to that reported by other authors [38], does not allow relevant conclusions to be drawn, although the non-significant reduction in the activity of this enzyme in combination with those of AST and GGT indicates an improvement in the liver function of old hens after short-term addition (1 week) of OEO and the absence of OEO-dependent hepatic lesions. Similarly, a previous study [73] noted that an elevation in ALP and AST concentrations in serum associated with experimentally induced oxidative liver lesions in Wistar rats by carbon tetrachloride injection was reduced after dietary oregano but only with long-term administration (6 weeks). However, this parameter is not specific to the diagnosis of liver disease; it also indicates increased osteoblast and osteoclast activity (bone growth, egg laying, rickets, or osteomalacia) [35], indirectly exploring mineral metabolism in laying hens. Alkaline phosphatase is also produced by osteoblasts along with other proteins that regulate bone mineralization [57].

In addition to ALP, which does not vary significantly, the mineral profile established in this study mainly concerns changes in calcium and phosphorus concentrations and the ratio of these two parameters (Ca/P) after the addition of OEO. For calcium, the feed analysis data show that the ration is rich in calcium. Based on feed intake in each flock, high consumption of the calcium available in the ration delivered to both flocks F1 and F2 (7.03 g and 6.02 g, respectively) was recorded before OEO administration. This high-calcium intake and low demand for egg production (for shell formation), which remained low, especially in flock 2, were probably responsible for the high plasma calcium levels exceeding acceptable limits in both flocks before the addition of OEO. The decrease in the feed intake at the end of the OEO treatment was automatically accompanied by a decrease in the amount of calcium ingested by the hens in both the F1 and F2 groups (6.55 g and 5.84 g, respectively), but still exceeding the recommendations of 4.7 g/hen/day for hens over 77 weeks of production [32]. Based on plasma calcium balance, it can be suggested that the OEO regulated calcium levels by reducing its concentration in the blood of 65 mg/L at the end of the treatment week, indicating a significant beneficial effect of OEO. This decrease is similar to the dose-dependent decrease recorded when the diet was supplemented with a mixture of essential oils containing a phenolic compound, thymol, at a concentration of 100 mg EO/kg [68]. In contrast, an increase was obtained with the inclusion of 50 mg EO/kg containing thymol [68] or the inclusion of oregano herb at 20 g/kg in another study [42]. Other authors found no significant effect when 24 mg/kg was included in a study conducted on hens older than 82 weeks [40]. However, in these previous studies, the calcium levels in the rations, namely, 3.8% [42], 3.83% [68], and 3.94% [40], were lower than that of the ration distributed to the two flocks (5.4%). In the present study, the decrease in calcium concentrations after administration of OEO may reflect a mobilization of excess calcium from the blood to the shell, as reflected in the weight and percentage of the shell discussed above, probably via an improvement in the absorption of circulating calcium by the tubal (oviduct) and uterine membranes. This effect appears to be linked to a possible beneficial effect of OEO use in eliminating possible bacterial membrane and cell alterations [17,19,20,44,48,63] by virtue of the antimicrobial, antioxidant, and anti-inflammatory properties of OEO and its main phenolic compound, carvacrol. Regardless of the flock of origin and the age of the late-laying hens, plasma phosphorus levels showed a significant increase after administration of OEO while remaining within the reference ranges. This is consistent with the results recorded in Hy-Line Brown laying hens at 60 weeks of age that received 20 g of oregano herb per kg of diet containing 0.62% phosphorus [42], a percentage close to that of 0.65% in the present diet distributed to the two flocks. However, other authors have not found a significant effect following dietary OEO supplementation in hens at the end of their laying cycle [40] or in the youngest laying hens (48 weeks of age) after long-term diet supplementation with a mixture of encapsulated essential oils comprising thymol and other components at different levels of inclusion [68]. The balance and regulation (homeostasis) of the two minerals (Ca and P), which play an important role in eggshell formation and skeletal (bone) integrity, are influenced by dietary intake and hormonal control, in particular, by the parathyroid hormone (PTH), fibroblast growth factor 23 (FGF23) [74], and Vitamin D, which controls calcium absorption in the duodenum and jejunum [57,75]. This absorption is affected by the levels of phosphorus available in the diet; conversely, the mobilization of calcium from the bones, facilitated by osteoclasts, also releases phosphorus into the circulation [75]. The reverse OEO-induced kinetics of calcium, which decreases to normal levels, and phosphorus, which increases, were indicative of the balancing effect of OEO on the Ca/P ratio, which fell significantly from above normal levels to reach acceptable levels at the end of treatment, indicating a beneficial effect of OEO on the homeostasis of these two minerals. The OEO therefore optimized the transfer of calcium from the blood to the uterine fluid surrounding the outer membrane of the shell by exploiting the high blood flow in the uterus. Physiologically, the daily dietary intake of calcium covers only two-thirds of the amount transported to the egg, 100 mg/h to 200 mg/h [76] or even 300 mg/h [32], and the deficit is made up by the intense mobilization of bone calcium (medullary bone) during the 12–18 h following the laying of the previous egg [77]. To compensate for the efficiency of this mobilization of Ca ions, it has been assumed that the hen reduces her consumption so that the net transfer of calcium is zero during the ovulatory sequence [76]. The mobilization of calcium in the present study could therefore explain the decrease in the intake observed as a process of calcium metabolism adapting to the increase in the number of ovulatory periods (laying rates) noted following the addition of OEO.

The demonstration of this dietary regulation in response to OEO administration requires evaluation of the endocrine response of the hen by analyzing variations in plasma procalcitonin and estradiol levels before and at the end of treatment. As with all biochemical parameters, variations in these endocrine parameters are attributed uniquely to the effect of OEO administration without any other effects or factorial interactions. Procalcitonin is the precursor of calcitonin synthesis in chicken ultimobranchial glands [78]. Calcitonin works in conjunction with sex steroids (such as estradiol) and other systemic factors, including growth hormone, IGF-1, thyroid hormone, parathyroid hormone (PTH), and Vitamin D3, to support osteoblast proliferation and stimulate matrix production. Calcitonin regulates bone mineralization by reducing bone resorption, while PTH increases plasma calcium levels, and Vitamin D enhances calcium absorption in the intestines, all contributing to the control of plasma calcium levels [57]. Beyond this complexity in the regulation of mineral and bone metabolism, procalcitonin was used in some veterinary species as a marker of inflammation and infection [29]. In humans, it was associated with bacterial infection when its concentration increased in the blood, but it is less sensitive to viral and fungal infections [79]. In healthy humans, PCT is produced in thyroid C cells. Its elevation is mediated by cytokines and bacterial endotoxins during inflammation, bacterial infection, and sepsis. Some studies have suggested that several specific tissues or organs, such as the intestine, liver, pancreas, lungs, pituitary gland, and hypothalamus, may be the source of PCT in unhealthy individuals [80]. Motivated by its effectiveness in human medicine for identifying bacterial infections and evaluating antibiotic therapy [27,28], this parameter was included in this study to assess the anti-inflammatory properties and antimicrobial effectiveness of OEO on potential microbiota imbalances or bacterial infections, such as those caused by *Salmonella* [30]. The procalcitonin profile showed a significant decrease in concentrations when OEO was administered, without the influence of the “flock” factor or their interaction. Before treatment, the values observed in flock 1 and flock 2 (132.43 pg/mL and 142.67 pg/mL, respectively), were quite similar to those documented in the intestinal inflammatory response caused by *Clostridium perfringens* in broiler chickens (approximately 140 pg/mL) when compared to the control group that received a basal diet without exposure to *C. perfringens* (around 110 pg/mL) [31]. However, pre-treatment PCT values were lower than those obtained after intestinal lesions in broiler chickens exposed to *Salmonella enterica* serovar *Typhimurium* (249.07 pg/mL) versus those fed a basic diet without *S. typhimurium* challenges (199.36 pg/mL) [30]. The results of studies conducted on broiler chickens [30,31] showed a difference of 30 pg/mL and 50 pg/mL between chickens with and without bacterial intestinal inflammation (*Clostridia* and *Salmonella*, respectively). This suggests that the difference of 55 pg/mL in PCT inflammatory parameter concentrations before and after OEO administration could confirm an anti-inflammatory effect of its main compound (carvacrol) on the intestinal inflammatory process likely present in both flocks. Prior to OEO addition, this process appeared to be more pronounced in flock 2, which had a lower egg production rate, moderate medical history, and high PCT concentrations (142.67 pg/mL). Hens in this flock also had high pre-treatment liver enzyme levels, namely, GGT of 47.00 IU/L, exceeding the maximum limit of 25 IU/L, AST of 185.84 IU/L, within accepted limits, and ALP (528 IU/L) exceeding the average of 482.5 IU/L described by Kaneko et al. [36]. Plasma levels of GGT and AST, indicators of stress, hepatocyte damage, or both, showed a positive, albeit moderate, correlation with PCT. This relationship was illustrated by a decrease in the concentration of these enzymes alongside a reduction in PCT levels following OEO supplementation. This suggests an improvement in liver health and function, as well as an anti-inflammatory intestinal response. These changes are related to the possible improvement in metabolite absorption and the immune response, potentially dependent on the above-mentioned effects of carvacrol (antioxidant, antimicrobial, anti-inflammatory, hepatoprotective, and immunostimulant). The decrease in procalcitonin in response to this aromatherapy does not appear to act directly on calcium levels via the use of this precursor in calcitonin synthesis. This is justified by the absence of a significant correlation between calcium concentration and PCT. In contrast, OEO apparently exerted its effect by improving uterine absorption, correcting bacterial alterations in the membranes of the shell gland responsible for shell formation [57].

A remarkable rise in circulating estradiol levels in both flocks after the addition of OEO appears to be related to an improvement in ovarian function in hens in the final phase of their production cycle, regardless of their age in weeks. Estrogen synthesis occurs in theca cells of developing ovarian follicles, unlike mammals [81]. It is stimulated by gonadotropic hormones, follicle-stimulating hormone (FSH) and luteinizing hormone (LH), in response to hypothalamic gonadotropin-releasing hormone (GnRH) [82]. The primary substrate for steroidogenesis is cholesterol, which may affect follicle selection regulated by the hypothalamic–pituitary–gonadal axis [81]. It is known that the source of blood cholesterol is mainly the liver and diet [57,82]. The increase in estradiol production appears to be a marker of improvement, OEO- or carvacrol-dependent, in various metabolism (protein, hepatic, mineral) and immune status types, leading to the improvement in production performance noted above. The increase in plasma estradiol levels after OEO treatment, along with a decrease in plasma AST levels (negative correlation), reflects the direct interaction between the two functions: hepatic and ovarian. A recovery of liver cells, evidenced by a decrease in AST following OEO addition, ensures greater surface area and better expression of estrogen receptors, allowing estrogens to stimulate the synthesis of liver proteins to be transferred to the formation of egg yolk and for the transport of steroids, such as estradiol, as in the case of albumin [60,61]. The high demand for albumin in response to increased estradiol (transport and synthesis of egg yolk) explains the negative correlation between increased estradiol synthesis and decreased free plasma albumin concentrations observed in this study. In return, improved synthesis of the liver growth factor, insulin-like growth factor (IGF-I), together with growth hormone (GH), stimulates follicular growth and their steroid synthesis of estrogens [83] from lipids (cholesterol), also derived from hepatic metabolism [57,64,65]. The absence of a significant correlation between energy parameters (glucose, cholesterol, and triglycerides) and the increase in estradiol at the end of OEO administration does not exclude an effect of energy substrates. This is particularly evident for glucose, which acts on thecal synthesis of steroid hormones through the upregulation of key genes and proteins in the CREB/StAR (Cyclic AMP Responsive Element-Binding protein/Steroidogenic Acute Regulatory) signaling pathways found in laying hens [84]. Moreover, a logical improvement in ovarian function (follicular growth) is indicated by an increase in E2 levels, which, together with growth factors, bind to their specific receptors present in the shell gland (ER-α, GH-R, and IGF-IR) [85]. As a result, egg production increases following the administration of OEO, leading to increased use of blood calcium in the shell calcification process. These findings are confirmed by a significant negative correlation between the decrease in the Ca/P ratio and the increase in estradiol, thus demonstrating the role of this steroid hormone in bone calcium metabolism through the estrogen receptor [86]. The involvement of the hypothalamic–pituitary axis in explaining the improvement in estradiol levels after OEO supplementation should be considered [82,86], given that high PCT levels may also originate specifically in the hypothalamus and pituitary gland, as noted in humans [80]. Despite the absence of a significant correlation between estradiol and PCT levels, the decrease in the latter inflammatory parameter after treatment may indicate an improvement in the functioning of the liver, hypothalamic–pituitary–ovary axis, and uterus. However, extending the hormonal profile to include gonadotropic hormones (FSH and LH) could have provided more information about the relationship between immunity (PCT), metabolism (metabolites), and the reproductive axis (estradiol). Oregano, such as *Origanium vulgare*, contains significant phytoestrogens in its hydroalcoholic extract, including luteolin-glucoside and luteolin- and apigenin-glucuronides [87,88]. In mammals, these phytoestrogens can act on estrogen receptors and stimulate oocytes and pituitary gonadotropic cells [89]. Dietary supplementation with *Origanum majorana* powder as a source of phytoestrogens increased FSH, LH, and estradiol in 45-week-old Bovans Brown laying hens when combined with *Salvia officinalis*. These phytoestrogenic plants had a positive effect on productive performance, ovarian activity, reproductive hormones, and antioxidant activity during the post-peak laying period in hens [90]. However, in the present study, estrogen-like or estrogen-mimetic flavonoids are a non-volatile hydroalcoholic extract and are therefore not present in oregano essential oil, which mainly contains volatile components commonly extracted by hydrodistillation. Thus, the stimulating effect of OEO therapy on estradiol levels does not appear to be directly related to the flavonoids commonly found in the hydroalcoholic extract of the plant but could be linked to properties of the main volatile phenolic component of OEO (carvacrol) on the complex of endocrine-regulated structures comprising the liver, the hypothalamic–pituitary–gonadal axis, and the shell gland. The antioxidants present in OEO, via their neutralizing effect on reactive oxygen species (ROS), can reduce the risk of damage to follicular cells, particularly thecal steroidogenic cells, thereby improving estrogen levels. A stimulating effect on follicular growth and, consequently, on estrogen synthesis, probably occurs by virtue of the hepatoprotective action of phenolic compounds on the liver, ensuring adequate levels of IGF, one of the main modulators of this growth. The anti-inflammatory and antibacterial effects of these OEO compounds may act on the various structures of the genital tract, particularly the ovaries and uterus, thereby improving the mechanism that controls circulating estrogen levels, which is also linked to the level of calcium absorbed. Increased substrate availability through improved intestinal absorption and hepatocyte function, as well as normalization of lipid metabolism, including cholesterol, a precursor to estrogen synthesis, may be associated with the bioactive properties of carvacrol. In addition, the anti-inflammatory properties of OEOs could modulate the hypothalamic–pituitary–ovarian axis by reducing inflammatory signaling, associated with a decrease in PCT levels, as inflammatory cytokines can affect GnRH secretion and, consequently, gonadotropins that stimulate sex steroid synthesis. These hypotheses suggest that further research should be conducted to assess whether gonadotropin levels (FSH, LH), hepatic estrogen receptor expression, ovarian steroidogenic enzyme activity, and hypothalamic inflammatory markers could change simultaneously with sex hormone production in response to the effects of essential oil components.

## 5. Conclusions

This real-world study focused on evaluating metabolic and endocrine profiles alongside performance metrics to assess the effectiveness of a short-term addition of a marketed oregano essential oil in laying hens raised under commercial farming conditions. The results allow for developing hypotheses about the effects of this oil, via its main phenolic compound, carvacrol, on the possible modulation of the intestinal microbiota, improved immunity and antibacterial activity, and control of inflammation and oxidative stress in organs (liver and genital tract), which is particularly important in hens at the end of their productive cycle. The OEO probably exerted its effect by improving absorption mechanisms, correcting any bacterial alterations, mainly in the intestine and secondarily in the liver, ovaries, oviduct, and shell gland, potentially related to a decrease in plasma procalcitonin levels. These effects were also associated with an increase in the circulating proteins necessary for immunoglobulin synthesis, egg yolk formation, and the transport of metabolites and hormones. In addition, this supplementation appears to be linked to improved liver function through a reduction in liver enzymes and stimulation of the ovarian activity associated with better estrogen synthesis. The increase in this sex hormone could improve bone mineral metabolism in terms of the observed normalization of the calcium–phosphorus balance. Although OEO administration was followed by a significant increase in egg production rates in older hens and was associated with enhancements in feed efficiency in both flocks, enhanced egg weight in young hens, and an inconsistent reduction in mortality, variations in production performance remain multifactorial and cannot be attributed solely to this additive. It appears that hens modify their production abilities as a pathway of adaptation to better homeostasis. This process, which aims to stabilize the physiological constants of the hens and maintain them within a normal range, involves homeorhesis mechanisms that engage the intestinal microbiota, liver, bones, reproductive axis, and shell gland. Analysis of blood test data suggests that OEO has a reinforcing effect on the adaptive potential of hens in the critical phase of production. However, in the absence of a control group linked to the on-farm condition, these suggestions require further investigation of other blood parameters, such as antioxidant enzymes, metabolic regulatory hormones, such as insulin growth factors, and reproductive hormones, such as gonadotropic hormones and progesterone, as well as the study of the hepatic estrogen receptor. Overall, these findings highlight both the economic and pharmacological benefits of *Origanum* essential oil as a versatile natural antimicrobial additive and report, for the first time, the relevance of using procalcitonin to assess the effectiveness of this nutraceutical ingredient in laying hens. However, further research will be necessary to deepen its pharmacodynamic and toxicological evaluation in order to maximize zootechnical benefits while preserving animal welfare and food safety.

## Figures and Tables

**Table 1:** Ingredients and chemical composition of the distributed laying hen diet in the two monitored flocks.

Ingredients	Proportion (%)
Corn	62
Soybean	19
Wheat bran	8
Grit	8
Monocalcium phosphate *	1.5
Vitamin and mineral premix **	1
Total	100
**Analytical nutrient composition (per kg of diet)**	
Dry matter (DM)	91.4
Crude protein (CP)	19
Ether extract (Fat)	2.8
Sugar	50.2
Crude fiber (CF)	4.5
Ash	13.7
Calcium	5.4
Phosphorus	0.65
Metabolizable energy (kcal/kg)	2734

*: Monocalcium phosphate content: total phosphorus 22.7%, calcium 15%. **: Premix provided per kg of diet: Vitamins = Vitamin A 10,000 IU, Vitamin D3 3000 IU, Vitamin E 20 mg, Vitamin B1 2 mg, Vitamin K3 2 mg, Vitamin B2 6 mg, Vitamin B6 4 mg, Vitamin B12 0.02 mg, Niacin 30 mg, Pantothenic acid 10 mg, Biotin 0.1 mg, and Folic acid 1 mg. Oligo-elements = Iron 50 mg, Copper 6.25 mg, Zinc 50 mg, Manganese 87.50 mg, Iodine 1.25 mg, Cobalt 0.50 mg, and Selenium 0.25 mg. Additives = DL-Methionine 1200 mg, Choline chloride 400 mg, Antioxidant 100 mg, Salt 3000 mg, calcium 11.40%, Sodium 13%, and Chlorine 19%.

**Table 2:** Effect of oregano essential oil oral therapy on egg quality of Hy-Line Brown laying hens in the two studied flocks.

Flocks		Flock1			Flock2		
Treatment Periods		Pre-OEO Tx	Tx-OEO Week	Post-OEO Tx Week	Pre-OEO Tx	OEO Tx-Week	Post-OEO Tx Week
Hens Age (Weeks)		96 (W0)	97 (W1)	98 (W2)	79 (W0)	80 (W1)	81 (W2)
Average Egg Weight (g)	*n*	5	5	5	5	5	5
	Mean	67.62 ^a^	70.44 ^a^	68.44 ^a^	61.66 ^a^	71.38 ^b^	69.86 ^b^
	SEM	3.82	4.45	3.45	1.82	1.08	3.68
	*p*_W0 vs. W1_	0.621			0.016		
	*p*_W1 vs. W2_	0.725			0.669		
	*p*_W0 vs. W2_	0.885			0.036		
	Standard values	65.4	65.4	65.4	65	65	65
Eggshell Weight (g)	*n*	5	5	5	5	5	5
	Mean	8.80 ^a^	10.00 ^a^	9.50 ^a^	8.62 ^a,b^	8.22 ^b^	10.04 ^a^
	SEM	0.51	0.38	0.29	0.65	0.38	0.40
	*p*_W0 vs. W1_	0.057			0.575		
	*p*_W1 vs. W2_	0.397			0.022		
	*p*_W0 vs. W2_	0.243			0.063		
Eggshell Ratio (%)	*n*	5	5	5	5	5	5
	Mean	13.04 ^a^	14.39 ^a^	14.05 ^a^	13.94 ^a^	11.51 ^b^	14.44 ^a^
	SEM	0.43	0.93	0.91	0.84	0.45	0.44
	*p*_W0 vs. W1_	0.251			0.015		
	*p*_W1 vs. W2_	0.767			0.005		
	*p*_W0 vs. W2_	0.385			0.577		
	Reference values *	10–11	10–11	10–11	10–11	10–11	10–11

^ab^ For each flock, mean values with different superscripts in the same row are significantly different at *p* < 0.05 using the LSD test. Standard values were retrieved from the Hy-Line International management guide [32]. * Reference values for eggshell percentage cited by Gautron et al. [33].

**Table 3:** GLM analysis of biochemical parameter concentrations (mean ± SEM) measured in the two flocks of laying hens before (Pre-OEO Tx) and after (Post-OEO Tx) receiving oregano essential oil.

	Flocks				Total Treatment		Fixed Effects						Reference Value (Range [35]; Mean [36])
	Flock 1		Flock 2				Flock		Treatment		Flock * Treatment		
	Pre-OEO Tx	Post-OEO Tx	Pre-OEO Tx	Post-OEO Tx	Pre-OEO Tx	Post-OEO Tx	F	Sig.	F	Sig.	F	Sig.	
n	10	10	10	10	20	20							
TP (g/L)	54.69	63.68	53.93	57.84	54.31	60.76	2.40	0.13	9.20	0.01	1.43	0.24	43.8–68.6; 56
	±2.15	±2.01	±2.32	±2.01	±1.58	±1.42							
Alb (g/L)	21.10	20.41	21.47	18.40	21.28	19.41	1.07	0.31	5.57	0.03	2.24	0.15	22.3–28.0; 25
	±0.80	±0.75	±0.87	±0.75	±0.59	±0.53							
Glob (g/L)	33.59	43.26	32.47	39.44	33.03	41.35	2.24	0.15	25.41	0.00	0.67	0.42	21.5–42.0; 31
	±1.67	±1.56	±1.80	±1.56	±1.23	±1.10							
Alb/Glob	0.64	0.48	0.67	0.47	0.65	0.47	0.12	0.74	41.29	0.00	0.37	0.55	0.64–1.1; 0.81
	±0.03	±0.03	±0.03	±0.03	±0.02	±0.02							
Glc (g/L)	2.51	2.28	2.37	2.53	2.44	2.41	0.22	0.64	0.10	0.75	3.14	0.09	1.91–3.42; 1.68
	±0.11	±0.11	±0.12	±0.11	±0.08	±0.08							
TG (g/L)	19.27	17.70	27.93	12.26	23.60	14.98	0.11	0.75	3.05	0.09	2.04	0.17	3.19–32.24
	±4.99	±4.67	±5.39	±4.67	±3.67	±3.30							
Chol (g/L)	1.56	1.54	1.84	1.52	1.70	1.53	0.18	0.67	0.33	0.57	0.25	0.62	1.08–2.93; 1.84
	±0.30	±0.29	±0.33	±0.29	±0.22	±0.20							
AST (UI/L)	190.96	140.13	185.84	114.96	188.40	127.54	1.05	0.32	16.94	0.00	0.46	0.50	130–270; 174.8
	±14.94	±13.98	±16.14	±13.98	±11.00	±9.88							
GGT (UI/L)	25.80	26.57	47.00	28.93	36.40	27.75	2.67	0.12	1.44	0.24	1.71	0.20	5–25
	±8.09	±6.83	±7.38	±6.39	±5.47	±4.68							
ALP (UI/L)	395.00	263.63	528.00	344.13	461.50	303.88	1.22	0.28	2.66	0.12	0.07	0.79	155–990; 482.5
	±97.59	±91.29	±105.41	±91.29	±71.83	±64.55							
Ca (mg/L)	386.43	362.88	416.00	307.00	401.21	334.94	0.29	0.60	7.35	0.01	3.06	0.09	132.26–360.72; 284
	±24.70	±23.10	±26.68	±23.10	±18.18	±16.34							
P (mg/L)	74.19	90.96	72.80	78.44	73.49	84.70	1.98	0.17	5.15	0.03	1.27	0.27	46.5–86.7
	±4.99	±4.67	±5.39	±4.67	±3.67	±3.30							
Ca/P	5.31	3.99	5.83	3.90	5.57	3.94	0.46	0.51	27.50	0.00	0.97	0.33	1.6–4.9
	±0.31	±0.29	±0.34	±0.29	±0.23	±0.21							

* Sig.: *p*-value of F-test for fixed effects, which are significant when *p* < 0.05. Total protein (TP), albumin (Alb), total globulin (Glob), glucose (Glc), triglycerides, total cholesterol (TC), aspartate aminotransferase (AST), gamma glutamyl transferase (GGT), alkaline phosphatase (ALP), calcium (Ca), phosphorus (P).

**Table 4:** Effects of oregano essential oil on specific biomarkers of bacterial infection (procalcitonin) and ovarian function (estradiol) and their relationship with the other biochemical parameters.

			Flocks				Total Treatment		Fixed Effects					
			Flock 1		Flock 2				Flock		Treatment		Flock * Treatment	
			Pre-OEO Tx	Post-OEO Tx	Pre-OEO Tx	Post-OEO Tx	Pre-OEO Tx	Post-OEO Tx	F	Sig.	F	Sig.	F	Sig.
PCT (pg/mL)		*n*	10	10	10	10	20	20						
		Mean	132.43	65.63	142.67	58.63	137.55	62.13	0.00	0.95	7.26	0.01	0.09	0.76
		SEM	28.29	26.46	30.56	26.46	20.82	18.71						
E_2_ (pg/mL)		n	10	10	10	10	20	20						
		Mean	1228.4	1406.9	882.3	1728.6	1055.4	1567.8	0.01	0.94	8.7	0.01	3.69	0.07
		SEM	175.6	164.3	189.7	164.3	129.2	116.1						
**Pearson correlation «r»**														
	E_2_	TP	Alb	Glob	Alb/Glob	Glc	TG	Chol	AST	GGT	ALP	Ca	P	Ca/P
PCT	0.13	−0.19	0.3	−0.24	0.35	−0.07	0.19	0.11	0.41	0.58	0.003	0.16	0.08	0.06
*p* value	0.51	0.54	0.11	0.20	0.06	0.72	0.34	0.57	0.03	0.002	0.99	0.4	0.69	0.75
	PCT	TP	Alb	Glob	Alb/Glob	Glc	TG	Chol	AST	GGT	ALP	Ca	P	Ca/P
E_2_	0.13	−0.02	−0.35	0.112	−0.32	−0.03	−0.21	−0.07	−0.42	0.012	−0.24	−0.3	0.16	−0.43
*p* value	0.51	0.91	0.03	0.49	0.04	0.85	0.2	0.66	0.007	0.95	0.14	0.06	0.32	0.005

* Sig.: *p* value of F-test for fixed effects, which are significant when *p* < 0.05. Procalcitonin (PCT), estradiol (E_2_), total protein (TP), albumin (Alb), total globulin (Glob), glucose (Glc), triglycerides, total cholesterol (TC), aspartate aminotransferase (AST), gamma glutamyl transferase (GGT), alkaline phosphatase (ALP), calcium (Ca), phosphorus (P).

## Data Availability

The original contributions presented in this study are included in the article. Further inquiries can be directed to the corresponding author(s).

## Abbreviations

The following abbreviations are used in this manuscript:

AEM	Average Egg Weight
Alb	Albumin
ALP	Alkaline Phosphatase
ALT	Alanine Transaminase
AST	Aspartate Aminotransferase
BW	Body Weight
Ca	Calcium
CM	Cumulative Mortality
CREB	Cyclic AMP Responsive Element-Binding protein
DM	Daily Mortality
E_2_	Estradiol
ESW	Eggshell Weight
FCR	Feed Conversion Ratio
FGF23	Fibroblast Growth Factor 23
FI	Feed Intake
FSH	Follicle-Stimulating Hormone
GGT	Gamma glutamyl transferase
GH	Growth Hormone
Glc	Glucose
Glob	Total Globulin
GLM	General Model Analysis
GnRH	Gonadotropin-Releasing Hormone
HDEP	Hen-Day Egg Production
IGF-I	Insulin-Like Growth Factor
LDH	Lactate Dehydrogenase
LH	Luteinizing Hormone
OEO	Oregano Essential Oil
P	Phosphorus
PCT	Procalcitonin
PTH	Parathyroid Hormone
StAR	Steroidogenic Acute Regulatory
TC	Total Cholesterol
TGs	Triglycerides
TP	Total Protein
VLDL II	Apolipoprotein
VTG II	Vitellogenin
W	Week
